# Electron–phonon coupling in hybrid lead halide perovskites

**DOI:** 10.1038/ncomms11755

**Published:** 2016-05-26

**Authors:** Adam D. Wright, Carla Verdi, Rebecca L. Milot, Giles E. Eperon, Miguel A. Pérez-Osorio, Henry J. Snaith, Feliciano Giustino, Michael B. Johnston, Laura M. Herz

**Affiliations:** 1 Clarendon Laboratory, Department of Physics, University of Oxford, Parks Road, Oxford OX1 3PU, UK; 2 Department of Materials, University of Oxford, Parks Road, Oxford OX1 3PH, UK

## Abstract

Phonon scattering limits charge-carrier mobilities and governs emission line broadening in hybrid metal halide perovskites. Establishing how charge carriers interact with phonons in these materials is therefore essential for the development of high-efficiency perovskite photovoltaics and low-cost lasers. Here we investigate the temperature dependence of emission line broadening in the four commonly studied formamidinium and methylammonium perovskites, HC(NH_2_)_2_PbI_3_, HC(NH_2_)_2_PbBr_3_, CH_3_NH_3_PbI_3_ and CH_3_NH_3_PbBr_3_, and discover that scattering from longitudinal optical phonons via the Fröhlich interaction is the dominant source of electron–phonon coupling near room temperature, with scattering off acoustic phonons negligible. We determine energies for the interacting longitudinal optical phonon modes to be 11.5 and 15.3 meV, and Fröhlich coupling constants of ∼40 and 60 meV for the lead iodide and bromide perovskites, respectively. Our findings correlate well with first-principles calculations based on many-body perturbation theory, which underlines the suitability of an electronic band-structure picture for describing charge carriers in hybrid perovskites.

## Abstract (web summary)

Phonon scattering limits charge transport in perovskite solar cells, yet the interactions involved are still poorly understood. Here, Wright *et al*. show by photoluminescence measurements and first-principles calculations that longitudinal optical phonons dominate the electron-phonon coupling at room temperature.

## Graphical abstract

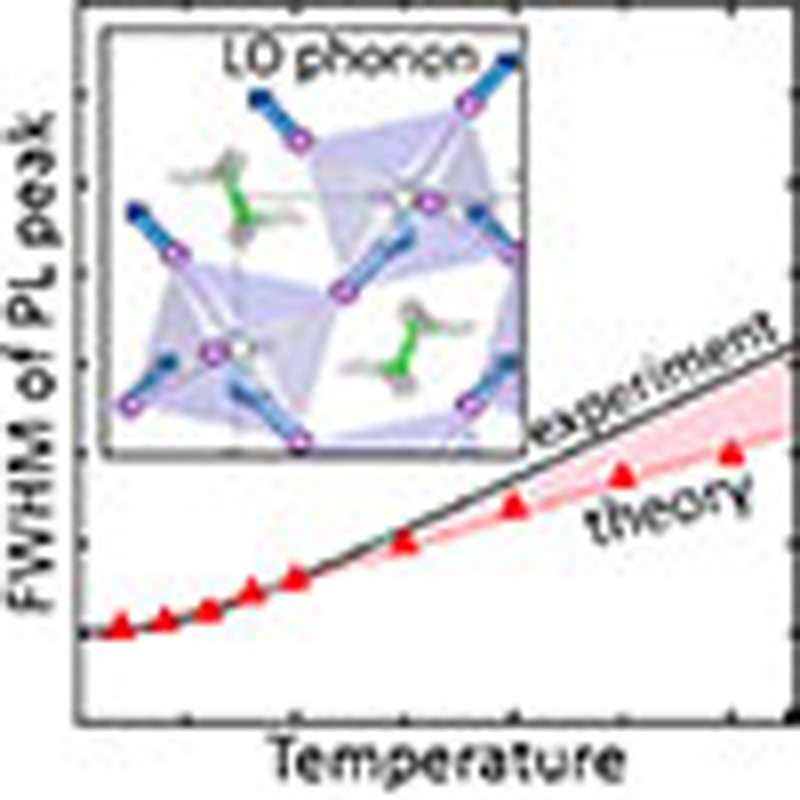

Hybrid lead halide perovskites have attracted intense research activity following their first implementation as light absorbers in thin-film solar cells^1^ that now reach power conversion efficiencies (PCEs) in excess of 20% (refs 2, 3). These compounds are described by the general formula ABX_3_, where A is typically an organic cation such as methylammonium (CH_3_NH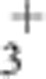 or MA^+^) or formamidinium (HC(NH_2_)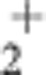 or FA^+^), B is a divalent metal cation (usually Pb^2+^) and X is a halide anion (I^−^, Br^−^ or Cl^−^)^4^. Such hybrid organic–inorganic materials straddle the divide between organic and inorganic semiconductors, facilitating photovoltaic devices that combine the low processing costs of the former with the high PCEs of the latter^2^,^3^,^5^. In addition, their structural flexibility allows a wide compositional parameter space to be explored. Although MAPbI_3_ is the most commonly investigated perovskite, the currently most efficient perovskite solar cells replace all^2^ or most^3^ of the MA^+^ with FA^+^. FAPbI_3_ has the advantage of a smaller bandgap than MAPbI_3_ (1.48 versus 1.57 eV), making it more suited for use in single-junction solar cells^6^, and furthermore shows greater resistance to heat stress^6^. High-performing devices have used formulations containing both FA and MA cations, to counteract the thermodynamic instability of FAPbI_3_ in its perovskite phase at room temperature^7^,^8^,^9^. Meanwhile, mixed-halide perovskites incorporating both I^−^ and Br^−^ have been investigated for use in tandem solar cells^5^,^6^,^10^, as these systems allow for bandgap optimization across a wide tuning range.

The success of hybrid perovskites in photovoltaic applications has been widely attributed to their high absorption coefficients across the visible spectrum^11^, their low exciton binding energies^4^,^12^ facilitating charge formation and their long charge-carrier diffusion lengths enabling efficient charge extraction^13^,^14^. However, although much recent attention has been devoted towards unravelling the charge-carrier recombination mechanisms underlying these properties^4^,^15^, the interaction of charge carriers with lattice vibrations (phonons) is currently still a subject of intense debate^16^,^17^. Such electron–phonon interactions matter, because they set a fundamental intrinsic limit to charge-carrier mobilities in the absence of extrinsic scattering off impurities or interfaces^18^. In addition, charge-carrier cooling following non-resonant (above bandgap) photon absorption is governed by interactions between charges and phonons^4^. Slow charge-carrier cooling components (compared with GaAs) have been postulated for hybrid perovskites^19^, which may open the possibility for PCEs beyond the Shockley–Queisser limit. Furthermore, electron–phonon coupling has been shown to yield predominantly homogeneous emission line broadening in hybrid lead iodide perovskite at room temperature, making it suitable as a gain medium for short-pulse lasers^20^.

Despite the importance of electron–phonon interactions to the optoelectronic properties of these materials, currently no clear picture has emerged of which mechanisms are active. To address this issue, a number of studies have examined the temperature dependence of the charge-carrier mobility *μ*, which was found^21^,^22^,^23^,^24^ to scale with *T*^*m*^ with *m* in the range between −1.4 and −1.6. Several groups^16^,^17^,^24^ therefore proposed that electron–phonon coupling at room temperature is almost solely governed by deformation potential scattering with acoustic phonons, which is known^18^,^25^ to theoretically result in *μ*∝*T*^−3/2^. Although such behaviour may be adopted by non-polar inorganic semiconductors such as silicon or germanium^18^,^26^, it would be extremely unusual for perovskites that exhibit polar^27^,^28^ lead–iodide bonds. These findings have therefore raised the puzzling question of why such hybrid perovskites appear to evade the Fröhlich interactions between charge carriers and polar longitudinal optical (LO) phonon modes that normally govern polar inorganic semiconductors, such as GaAs^18^,^29^, at room temperature.

Here we clarify the relative activity of different charge-carrier scattering mechanisms in hybrid lead halide perovskites by investigating charge-carrier scattering through an analysis of the photoluminescence (PL) linewidth as a function of temperature between 10 and 370 K. By carefully examining the low-temperature regime in which thermal energies fall below those of high-energy LO phonon modes, we are able to clearly separate competing contributions from charge-carrier interactions with acoustic and optical phonons. We are therefore able to show unambiguously that Fröhlich coupling to LO phonons is the predominant cause of linewidth broadening in these materials at room temperature, with scattering from acoustic phonons and impurities being a minor component. We further demonstrate excellent agreement between the experimentally determined temperature dependence of the PL linewidth and theoretical values derived from *ab initio* calculations for MAPbI_3_. To elucidate how charge-carrier–phonon interaction strengths depend on perovskite composition, we examine FAPbI_3_, FAPbBr_3_, MAPbI_3_ and MAPbBr_3_, which represent a comprehensive set of the most commonly implemented organic and halide ingredients in hybrid perovskites. We show that although the choice of organic cation has relatively little effect on the Fröhlich interactions, bromide perovskites exhibit higher Fröhlich coupling than iodide perovskites as a result of their smaller high-frequency values of the dielectric function. Overall, our results conclusively demonstrate that electron–phonon coupling in hybrid lead halide perovskites follows a classic bandstructure picture for polar inorganic semiconductors, which are dominated by Fröhlich coupling between charge carriers and LO phonon modes in the high-temperature regime.

## Results

### Temperature-dependent PL spectra

**Figure 1:**
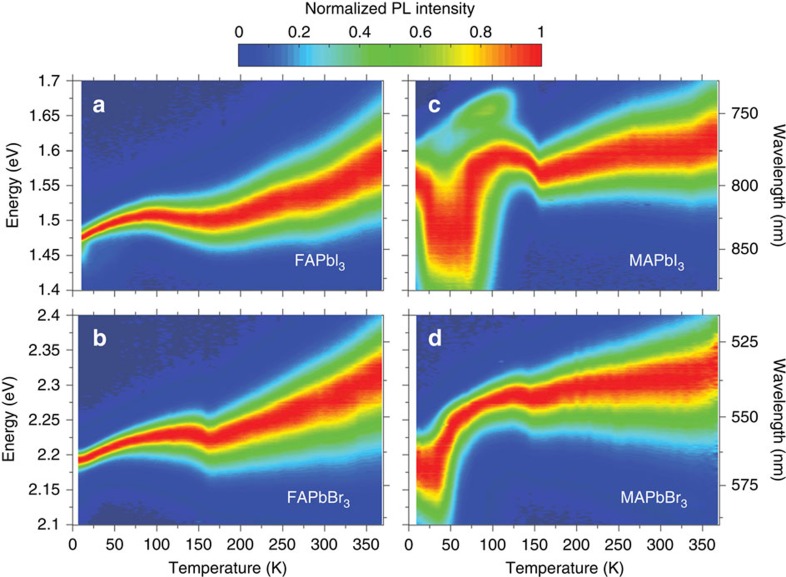
Temperature dependence of steady-state PL. Colour plots of normalized steady-state photoluminescence spectra of (**a**) FAPbI_3_, (**b**) FAPbBr_3_, (**c**) MAPbI_3_ and (**d**) MAPbBr_3_ thin films at temperatures between 10 and 370 K.

To conduct our analysis of electron–phonon coupling in hybrid lead halide perovskites, we recorded steady-state PL spectra of solution-processed FAPbI_3_, FAPbBr_3_, MAPbI_3_ and MAPbBr_3_ thin films over temperatures from 10 to 370 K in increments of 5 K (Fig. 1). The observed PL peak positions at room temperature are consistent with those reported previously for these materials^6^,^30^,^31^,^32^,^33^. The colour plots in Fig. 1 exhibit abrupt shifts in PL peak energies at various temperatures that are associated with phase transitions commonly found in these relatively soft materials. For example, MAPbI_3_, MAPbBr_3_ and FAPbI_3_ have been reported to undergo a phase transition from an orthorhombic to a tetragonal structure between 130 and 160 K (refs 30, 33, 34). A further phase transition to the cubic phase follows at higher temperatures for the MA perovskites (at ∼330 K for MAPbI_3_ (refs 30, 34) and 240 K for MAPbBr_3_ (ref. 34)), whereas a transition to a trigonal phase occurs at around 200 K for FAPbI_3_ (ref. 30). The lower-temperature phase transition to the orthorhombic phase below 130–160 K is generally associated with larger energetic shifts as it marks a strong reduction in the extent of rotational freedom of the organic cation^34^,^35^,^36^. Higher-temperature structural changes are more subtle in this regard and therefore much harder to discern^21^. Apart from these discontinuities, the PL peak in all four materials shifts continuously towards higher energy with increasing temperature. This general trend is in contrast to that of typical semiconductors such as Si, Ge and GaAs, for which the bandgap decreases with temperature as a result of lattice dilation^26^,^37^. The atypical positive bandgap deformation potential of hybrid lead halide perovskites has been attributed to a stabilization of out-of-phase band-edge states as the lattice expands^38^.

In addition to these well-understood temperature trends, the PL spectra of MA-containing perovskites exhibit strong inhomogeneous broadening and multi-peak emission in the low-temperature orthorhombic phase. Such behaviour has been reported by ourselves^21^,^39^ and others^24^,^31^,^40^,^41^ on many occasions, yet a precise explanation is still outstanding. For example, the PL spectra of MAPbI_3_ develop additional peaks at temperatures below 150 K, as can be seen clearly in Supplementary Fig. 1, which shows spectra from the colour plots in Fig. 1 at selected temperatures. The emerging consensus is that these are caused by additional charge or exciton trap states that are only active in the low-temperature orthorhombic phase^21^,^24^,^31^,^39^,^40^,^41^. It has been proposed that these traps could derive from a small fraction of inclusions of the room-temperature tetragonal phase that are populated through charge or exciton transfer from the majority orthorhombic phase^39^. These inclusions could be a result of strain or the proposed impossibility of a continuous structural transition from the tetragonal to orthorhombic phase in MA perovskites, as reported by Baikie *et al*.^35^. We further note that as these complicating features do not appear in the PL spectra of the equivalent FA perovskites, they are probably not intrinsic to hybrid lead halide perovskites, which re-affirms the prevailing view that they originate from trap states. Importantly, the lack of uncomplicated low-temperature spectra has to date prevented proper analysis of the linewidth broadening of MA perovskites and the associated phonon coupling to charge carriers. As we show below, such analysis requires access to a low-temperature range over which PL spectra are dominated by intrinsic phonon broadening, rather than trap-related PL, for the contributions from acoustic and optical phonons to the clearly separated. Therefore, the discovery that the equivalent FA lead halide perovskites do not exhibit extrinsic defect-related PL in the low temperature range allows us to carry out such analysis unhindered.

### Analysis of PL linewidth broadening

**Figure 2:**
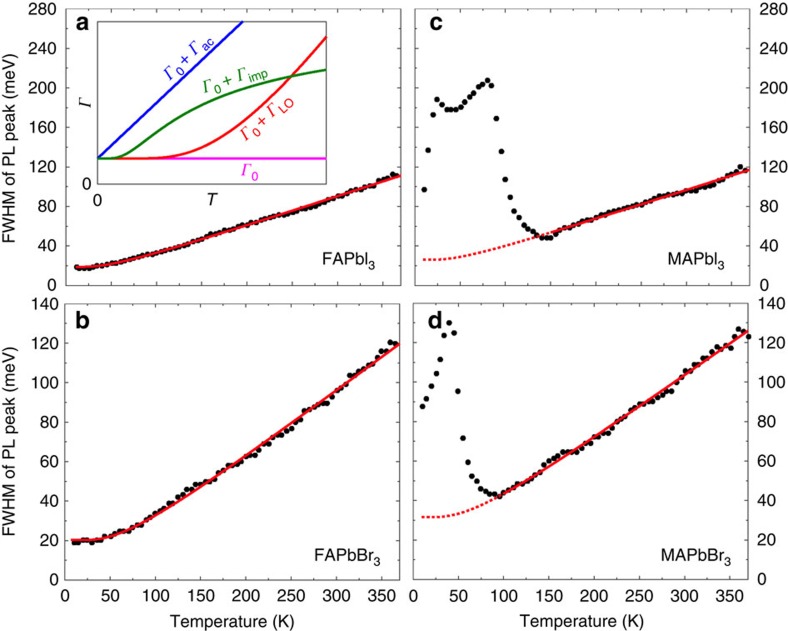
Temperature dependence of linewidth. FWHM of the steady-state PL spectra as a function of temperature for (**a**) FAPbI_3_, (**b**) FAPbBr_3_, (**c**) MAPbI_3_ and (**d**) MAPbBr_3_ thin films plotted as black dots. The solid red lines are fits of *Г*(*T*)=*Г*_0_+*Г*_LO_, which account for contributions from inhomogeneous broadening and Fröhlich coupling with LO phonons. For the perovskites containing MA, the fits are extrapolated into the low-temperature region in which the model does not hold, as indicated by dashed red lines (actual fits were carried out between 150 and 370 K for MAPbI_3_, and between 100 and 370 K for MAPbBr_3_). The inset shows the functional form of the temperature dependence of the contributions to PL linewidth in semiconductors from inhomogeneous broadening (*Г*_0_, magenta), Fröhlich coupling between charge carriers and LO phonons (*Г*_LO_, red) and acoustic phonons (*Г*_ac_, blue), and scattering from ionized impurities (*Г*_imp_, green), as given by the terms of equation (1). An alternative presentation of the linewidths as a mutliple of the thermal energy is given in Supplementary Fig. 2.

Analysis of temperature-dependent emission broadening has long been used to assess the mechanisms of electron–phonon coupling in a wide range of inorganic semiconductors^42^ (see Supplementary Table 1 for a literature overview of the results). We here apply these methods to hybrid lead halide perovskites by first extracting the full width at half-maximum (FWHM) of the PL spectra shown in Fig. 1 and then analysing its temperature dependence (plotted in Fig. 2). For most inorganic semiconductors, different mechanisms of scattering between charge carriers and phonons or impurities are associated with different functional dependencies of the PL linewidth *Г(T)* on temperature, which can be expressed as the sum over the various contributions^42^,^43^:


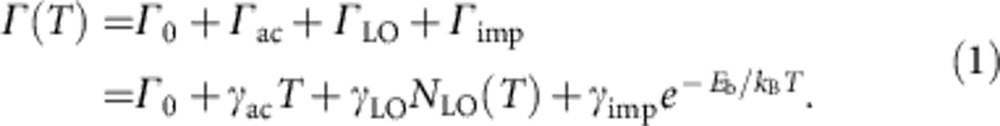


Here, *Г*_0_ is a temperature-independent inhomogeneous broadening term, which arises from scattering due to disorder and imperfections^42^,^44^. The second and third terms (*Г*_ac_ and *Г*_LO_) are homogeneous broadening terms, which result from acoustic and LO phonon (Fröhlich) scattering^28^,^42^,^44^ with charge-carrier–phonon coupling strengths of *γ*_ac_ and *γ*_LO_, respectively. Electron–phonon coupling is in general proportional to the occupation numbers of the respective phonons, as given by the Bose–Einstein distribution function^45^,^46^, taken as 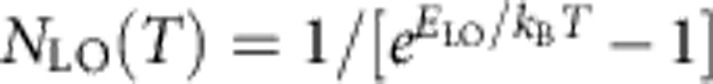 for LO phonons, where *E*_LO_ is an energy representative of the frequency for the weakly dispersive LO phonon branch^18^,^47^. For acoustic phonons whose energy is much smaller than *k*_B_*T* over the typical observation range, a linear dependence on temperature is generally assumed^46^,^48^. *Ab initio* calculations of the relevant phonon energies and occupation numbers are shown in Supplementary Fig. 4, which confirms that the linear approximation to the acoustic phonon population used in equation (1) is appropriate. The final term, *Г*_imp_, phenomenologically accounts for scattering from ionized impurities with an average binding energy *E*_b_ (ref. 43). These impurities contribute *γ*_imp_ of inhomogeneous broadening to the width when fully ionized^43^,^45^.

In general, the two major mechanisms governing the electron–phonon coupling in inorganic semiconductors are deformation potential scattering, in which distortions of the lattice change the electronic band structure, and electromechanical or piezoelectric interactions, in which lattice-related electric fields modify the electronic Hamiltonian^18^. Specifically, the LO phonon term in equation (1) accounts for the Fröhlich interaction between LO phonons and electrons, which arises from the Coulomb interaction between the electrons and the macroscopic electric field induced by the out-of-phase displacements of oppositely charged atoms caused by the LO phonon mode^18^. Although both transverse optical and LO phonons interact with electrons via non-polar deformation potentials, equation (1) only accounts for LO phonons because of the dominant influence of their Fröhlich interaction with electrons in polar crystals at higher temperatures^49^. As optical phonons in semiconductors typically have energies of the order of tens of meV (ref. 18), their population at low temperatures (*T*<100 K) is very small; thus, homogeneous broadening in this regime predominantly results from acoustic phonons^18^,^49^. Therefore, careful examination of the low-temperature regime allows separation of the contributions from optical and acoustic modes. Long-wavelength acoustic phonons induce atomic displacements, which can correspond to macroscopic crystal deformation, affecting electronic energies via either the resultant deformation potential or a piezoelectrically induced electric field^18^.

To establish qualitatively which electron–phonon scattering mechanisms contribute in hybrid lead halide perovskites, we compare the temperature-dependent PL linewidth plotted in Fig. 2 with the functional form of the terms in equation (1). To aid comparison, the inset to Fig. 2a shows example functions for the separate components. First, we assess the possibility of electron scattering with ionized impurities playing a significant role. Comparison of the curves in the inset with the data in the main Fig. 2 makes it apparent that the shape of the ionized impurity scattering term *Г*_imp_ could not produce the observed linear variation with *T* of the linewidths at high temperatures. Therefore, we conclude that scattering with ionized impurities does not play any major role here, in agreement with findings based on recent analyses of the temperature-dependence of the charge-carrier mobility in this regime^21^,^22^,^23^,^24^. We therefore assume *Г*_imp_≈0 for the rest of the analysis.

To separate the contributions from acoustic and optical phonon modes, we first focus on an analysis of the PL linewidth for perovskites containing FA as the organic cation. As Fig. 2a,b show, these materials exhibit smooth variation of the linewidth, whereas for MA-containing perovskites the presence of the impurity emission discussed above leads to additional emission broadening in the low-temperature phase (Fig. 2c,d). Both FAPbI_3_ and FAPbBr_3_ approach a PL linewidth of the order of 20 meV towards *T*=0, which can therefore be identified as the temperature-independent inhomogeneous broadening term *Г*_0_ arising from disorder. To qualitatively assess the relative importance of acoustic versus optical phonon contributions, an inspection of the gradient of these curves in the low-temperature regime is essential. Although the optical phonon terms lead to a gradient of zero in the regime for which *E*_LO_<*k*_B_*T*, the smaller energies of acoustic phonons should result in a non-zero gradient given by *γ*_ac_ here. However, visual inspection of the graphs in Fig. 2a,b shows that the gradient of the FWHM versus temperature approaches zero at low temperature, suggesting negligible acoustic phonon contribution (*γ*_ac_≈0). Indeed, we find that fits of equation (1) to these curves converge with *γ*_ac_→0. This result is not surprising, given that in polar inorganic semiconductors the contribution of acoustic phonons to the broadening at room temperature is typically dwarfed by that of the LO phonons and indeed several studies ignore the contribution of acoustic phonons in such systems^49^. However, we may obtain an upper limit to *γ*_ac_ by careful examination of the data in the low-temperature regime in which acoustic phonons are still expected to contribute significantly. Here we may fit *Г*(*T*)=*Г*_0_+*γ*_ac_*T* to the data in the low-temperature (*T*<60 K) region^50^ or, as an alternative method, obtain the gradient of the data near *T*=0 K from differentiation. Both methods yield upper limits around *γ*_ac_=60±20 μeV K^−1^ for FAPbI_3_ and FAPbBr_3_; therefore, acoustic phonons will only contribute up to ∼18 meV to the linewidth broadening at 300 K. Hence, our analysis illustrates that the majority of broadening in the room-temperature regime arises from charge-carrier interactions with optical phonons, as would be expected for a polar semiconductor.

To further quantify the dominant Fröhlich coupling in these systems, we proceed by fitting all data including only the mechanisms based on temperature-independent inhomogeneous broadening and Fröhlich coupling to LO phonon modes. For perovskites containing FA cations, fits of *Г*(*T*)=*Г*_0_+*Г*_LO_ to the PL linewidth data are plotted in Fig. 2a,b (red lines) and the extracted fitting parameters are presented in Table 1. Apart from Fröhlich coupling strengths, we are also able to determine the energy of LO phonon modes that play the dominant role in electron–LO-phonon coupling. We find *E*_LO_=11.5 meV for FAPbI_3_, with the value for FAPbBr_3_ (15.3 meV) being 1.3 times larger, which is only slightly greater than the factor of 1.2 expected from a crude model of the frequency of a diatomic harmonic oscillator. These LO phonon energies of hybrid lead halide perovskites are somewhat lower than those typically measured for a range of inorganic semiconductors (see Supplementary Table 1 for an overview); however, they agree well with a recent combined experimental and density functional theory (DFT) study assigning LO phonon modes of the Pb-I lattice in MAPbI_3_ with energies near 10 meV (ref. 51) and with our present calculations (see next section).

As noted above, the situation is more complex for MA perovskites, owing to the additional trap-related emission in the low-temperature orthorhombic phase that gives rise to sizeable (>100 meV) additional broadening (see Fig. 2c,d). However, in the high-temperature regime, PL linewidths of the MA perovskites vary much in the same manner as that of their FA counterparts, suggesting very similar mechanisms. This may be expected, as the organic cation has relatively little influence on the vibrations of the lead-halide lattice. Hence, we model the linewidth broadening of MA perovskites in the high-temperature regime again using *Г*(*T*)=*Г*_0_+*Г*_LO_ (solid red lines in Fig. 2c,d), using the LO phonon energies determined previously for FA perovskites. The resultant *γ*_LO_ values are, as expected, very similar to those for the corresponding FA perovskites (Table 1). We may also extrapolate these fits down through the low-temperature regime (dashed red lines), where they do not reflect experimental reality but rather show the broadening that would be present if the additional defects in the orthorhombic phase were absent. As such, values extracted for the parameter *Г*_0_ here mostly reflect inhomogeneous disorder present in the high-temperature phases of MAPbI_3_ and MAPbBr_3_ at temperatures above 150 and 100 K, respectively.

Finally, we comment on the extent to which excitonic effects may influence the coupling between charge carriers and phonons. Although the exact values for the exciton-binding energies in these systems are still a matter of debate, most reported values fall into the range of a few to a few tens of milli-electronvolts (see ref. 4 for a review). These values are compatible with numerous studies demonstrating that at room temperature, following non-resonant excitation, hybrid lead halide perovskites sustain free charge carriers as the predominant species^13^,^14^,^52^. Excitonic effects in the generated charge-carrier population are expected to increase as the temperature is lowered. However, we have recently examined infrared photoinduced transmission spectra for methylammonium lead iodide perovskite^21^ and found these to be predominantly governed by free-charge (Drude-like) features, with localization effects (for example, from excitons) only contributing at low temperature and not more than around 23% even at 8 K. This suggests that over the temperature window we examine here, emission broadening is mostly governed by interactions between phonons and free charge carriers rather than excitons. Such predominantly free-charge behaviour of the photogenerated species may be understood, for example, in terms of the Saha equation or a low Mott density for these systems, and considering that the initial highly non-resonant excitation generates predominantly free electron–hole pairs^12^. We may further inspect in more detail the temperature-dependent line shapes of the emission spectra and find these to exhibit high-energy Boltzmann tails corresponding to a thermalized electron–hole density near the lattice temperature (see Supplementary Fig. 3). These overall observations are therefore compatible with the presence of a thermalized free electron–hole charge-carrier density that scatters off mostly LO phonons whose occupancy is governed by the Bose–Einstein distribution function. Analysis of how the energies of such thermalized free electrons and holes influence the precise lineshape of the PL spectra as a function of temperature could provide additional insight into the scattering mechanisms for free charge carriers. Such additional analysis is however beyond the scope of the present investigation, which is limited to considering only the extent of linewidth broadening.

### First-principles calculations

**Figure 3:**
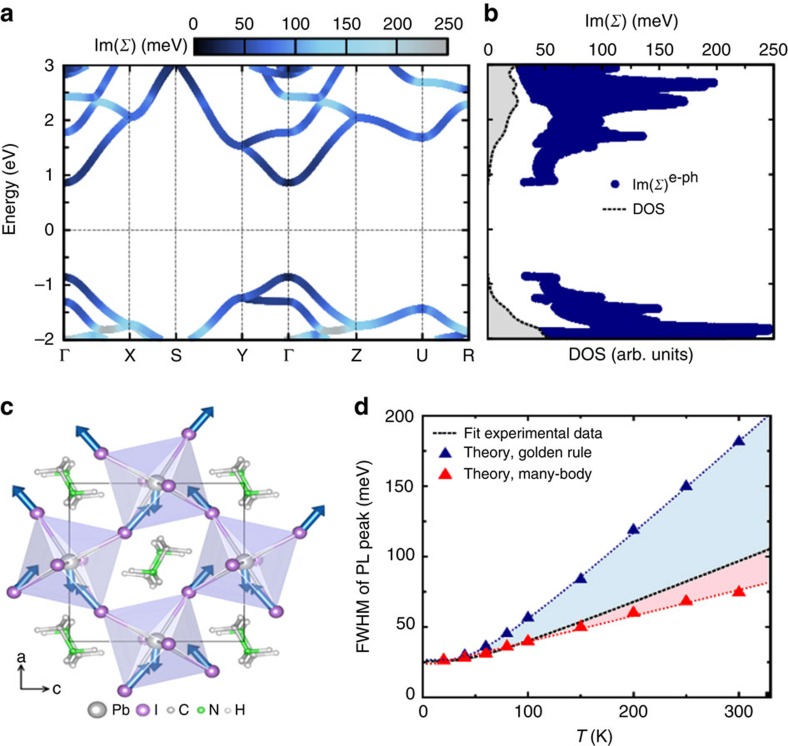
*Ab initio* calculations of electron–phonon coupling and PL broadening in MAPbI_3_. (**a**) Electronic band structure of orthorhombic MAPbI_3_, calculated within the GW approximation as in ref. 55, combined with a heat map of the imaginary part of the electron–phonon self-energy (Im(*Σ*)) at *T*=200 K. The zero of the energy is placed in the middle of the bandgap. In **b**, the imaginary part of the electron–phonon self-energy is shown together with the electronic density of states (DOS). 2 Im(*Σ*) corresponds to the electron/hole linewidth arising from electron–phonon coupling (apart from the quasiparticle renormalization factor *Z*). (**c**) Ball-and-stick representation of the LO vibration responsible for the broadening of the PL peaks. The blue arrows indicate the displacements of Pb and I atoms, for the phonon wavevector **q**→0 along the [100] direction. This is a Pb–I stretching mode with *B*_3*u*_ symmetry^51^. (**d**) Temperature dependence of the FWHM of the PL peak in MAPbI_3_: fit to the experimental data (dashed black line) and theoretical calculations using Fermi's golden rule (blue triangles) and the more accurate Brillouin–Wigner perturbation theory (red triangles). The theoretical broadening is obtained as the sum of 2 lm(*Σ*) at the valence and conduction band edges in the case of Fermi's golden rule, and the sum of 2 Z lm(*Σ*) when including many-body quasiparticle renormalization, rigidly shifted by the FWHM at *T*=0 K (25.72 meV), to account for inhomogenous broadening. The lines are guides to the eye.

We further corroborate our analysis with first-principles calculations of the electron–phonon coupling in MAPbI_3_ (see details in Methods section and in the Supplementary Note 1). In accordance with the above discussion, we separately consider the broadening arising from the interaction of phonons with free conduction-band electrons and free valence-band holes^53^. The combined broadening arising from both types of charge carrier is then compared with the experimentally determined emission broadening. In Fig. 3a, we present a heat-map of the imaginary part of the electron–phonon self-energy, Im(*Σ*), projected on the quasiparticle band structure of MAPbI_3_; 2 Im(*Σ*) represents the linewidth of electrons and holes arising from the electron–phonon interaction before accounting for many-body effects (see Methods) and is therefore directly comparable to the experimentally determined FWHM of the PL emission linewidth. Figure 3b shows Im(*Σ*) as a function of electron energy, together with the density of electronic states. This figure indicates that the increase in the linewidth is linked to the phase-space availability for electronic transitions, that is, Im(*Σ*) increases with increasing density of electronic states, because each state can scatter into a higher number of states by absorbing or emitting a phonon. Our calculations show that the dominant contribution to the electron–phonon self-energy arises from the coupling with the LO mode at 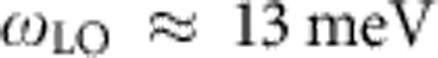, which is shown schematically in Fig. 3c. This observation is compatible with the analysis of the temperature dependence of the PL broadening, as presented in Fig. 2, yielding similar energy for the predominantly coupling LO phonon mode in MAPbI_3_.

Figure 3d shows that our calculated temperature dependence of the PL broadening is in good agreement with experiment. In this figure we compared our calculations with the experimental trends obtained from the fit shown in Fig. 2 (which does not account for the anomalous broadening below 150 K, as discussed above). The blue triangles represent the data calculated based on Fermi's golden rule, which is equivalent to using the imaginary part of the electron–phonon self-energy, 2 Im(*Σ*), whereas the red triangles were obtained by using Brillouin–Wigner perturbation theory^54^, which corresponds to scaling the self-energy by the quasiparticle renormalization factor *Z*, that is 2 *Z* Im(*Σ*) (see Methods). The comparison between calculations and experiments shows that the experimental data are most accurately described by fully taking into account the many-body renormalization of the electron lifetime. Taken together, the impressive agreement between (1) our measured and calculated characteristic phonon energy scale (11.5 and 13 meV, respectively), (2) the magnitude of our measured and calculated linewidth broadening at room temperature (90 and 75 meV, respectively), and (3) the phonon energy scale identified here and the LO phonon identified between 10 and 13 meV in our previous study^51^, strongly support the notion that the broadening of the PL spectra reflects the interaction between free carriers and LO phonons.

In addition, we may use first-principle calculations to elucidate why the lead bromide perovskites exhibit Fröhlich coupling constants that are larger than those of the lead iodide system by a factor of 1.5. To investigate this trend, we performed DFT calculations for MAPbBr_3_ (see Supplementary Note 1) to compare the associated Born effective charges between the two systems. Our results indicate that the electron–phonon coupling in the bromide perovskite is 40% stronger than in the iodide perovskite, in excellent agreement with our measurements. We find that the increased Fröhlich coupling in MAPbBr_3_ is primarily connected with the smaller high-frequency value of the dielectric function compared with MAPbI_3_.

## Discussion

Our analysis of the experimentally determined emission linewidth broadening and our first-principles calculations strongly support the notion that Fröhlich coupling to LO phonons is the predominant charge-carrier scattering mechanism in hybrid lead halide perovskites. As already discussed above, such behaviour is in many ways to be expected for these materials, because the lead-halide bond is sufficiently polar (see our calculated Born effective charges in Supplementary Table 2) to lead to macroscopic polarizations from LO phonon modes that modify the electronic energies, causing electron–phonon scattering. Coupling of charge carriers to phonons with energies in the meV range has also been postulated from the signature of such modes in the room- and low-temperature photoconductivity spectra^21^,^56^. However, the predominance of Fröhlich coupling appears at first sight to contradict the measured^21^,^22^,^23^,^24^ temperature dependence of the charge-carrier mobility, which has been stated^16^,^17^,^24^ to approach the expected form for acoustic deformation potential scattering (*μ*∝*T*^−3/2^). We note, however, that although electron–phonon coupling generally leads to charge-carrier mobilities that increase with decreasing temperature, the exact functional dependence is usually a composite of many different scattering mechanisms that can be hard to attribute uniquely^18^. Charge-carrier scattering with low-energy acoustic phonons can relatively easily be quantified by considering the distribution function of a nondegenerate electron gas approximated by a Boltzmann distribution, which gives the probability that a particular state with energy *E*_*k*_ is occupied at any temperature *T*. Such calculations result in predicted variations in mobility following *μ*∝*T*^−3/2^ for acoustic phonon deformation potential scattering^25^,^26^ and *μ*∝*T*^−1/2^ for acoustic phonon piezoelectric scattering^18^. For Fröhlich interactions between charges and LO phonons, analytical solutions are harder to establish^57^, but the reduction in LO phonon occupancy with decreasing temperature similarly leads to an increase in charge-carrier mobility in polar semiconductors^18^. Even for non-polar semiconductors such as silicon, electron–phonon interactions are found to be complex, for example, involving higher-order phonon terms and intervalley scattering^58^. Therefore, we conclude that although acoustic phonon deformation potential scattering may result in *μ*∝*T*^−3/2^, the converse may not necessarily also hold. In addition, the rapid energy loss of electrons observed following non-resonant excitation^19^,^59^ can only sensibly be explained by a succession of high-energy optical phonon emissions^60^, as is typically observed in inorganic semiconductors^4^,^61^. Therefore, we conclude that Fröhlich coupling to LO phonons, rather than acoustic phonon deformation potential coupling, is the dominant charge-carrier scattering mechanism at room temperature in these hybrid lead halide perovskites. Although our findings themselves do not explain the temperature dependence of the charge-carrier mobility in these materials, they support the hypothesis that the observed *μ*∝*T*^−3/2^ relationship is not wholly attributable to acoustic deformation potential scattering. It is also clear that for these high-quality materials, scattering from ionized impurities is a negligible component at room temperature, with both our PL linewidth data and earlier charge-carrier mobility measurements^21^,^22^,^23^, indicating a complete absence of such contributions.

In addition, our findings give early answers to the question of how perovskite composition affects Fröhlich interactions between charge carriers and phonons. Such interactions determine the maximum charge-carrier mobilities intrinsically attainable, which in turn affects charge-carrier extraction in solar cells. We show that Fröhlich coupling in hybrid lead bromide perovskites appears to be stronger because of the lower dielectric function in the high-frequency regime. Indeed, the THz charge-carrier mobility for FAPbBr_3_ thin films has recently been shown to be somewhat lower than that for FAPbI_3_ films^10^, which could be related to decreased momentum scattering time resulting from increased scattering with LO phonons. However, it may also be partly related to a higher propensity towards disorder in the bromide perovskites, as the inhomogeneous broadening parameter *Г*_0_ appears to be somewhat higher for bromide than iodide perovskites here. Similarly, higher Urbach energies have previously been reported for MAPbBr_3_ compared with MAPbI_3_, in accordance with larger energetic disorder in the former^62^. We also find that *Г*_0_ tends to be lower for the perovskites containing FA as the organic cation, which points to larger material uniformity as one reason behind this material's recent success in the highest efficiency perovskite solar cells^2^,^7^.

We may also compare these findings with those of other polar inorganic semiconductors for which Fröhlich coupling is known to be active. Supplementary Table 1 provides a detailed literature survey of values established for *γ*_LO_ in other inorganic semiconductors. It has been pointed out^17^ that charge-carrier mobilities established for lead halide perovskites (typically ≤100 cm^2^ (V s)^−1^)^15^,^17^ are relatively modest compared with those achieved in high-quality GaAs despite the effective charge-carrier masses in perovskites being only slightly elevated above those in GaAs. The values of *γ*_LO_≈40 meV and *γ*_LO_≈60 meV we extract from our data for the respective iodide and bromide perovskites (see Table 1) are somewhat higher than the range reported for GaAs (see comparison in Supplementary Table 1), which may partly explain these discrepancies. However, they are significantly lower than those typically found in highly polar materials such as GaN and ZnO where they can be over an order of magnitude higher^28^. Further theoretical modelling of charge-carrier mobility in these systems based on our findings will most probably allow more quantitative explanations and predictions to be made.

In summary, we have conducted an in-depth analysis of charge-carrier–phonon interactions in hybrid lead halide perovskites, considering the four currently most implemented organic and halide components in hybrid perovskite photovoltaics, which are FAPbI_3_, FAPbBr_3_, MAPbI_3_ and MAPbBr_3_. Our analysis of the temperature-dependent emission linewidth of FAPbI_3_ and FAPbBr_3_ allowed us to establish that the Fröhlich interaction between charge carriers and LO phonons provides the dominant contribution to the predominantly homogeneous linewidth broadening in these hybrid perovskites at room temperature. We successfully corroborated our findings with DFT and many-body perturbation theory calculations, which underline the suitability of an electronic bandstructure picture for describing charge carriers in perovskites. We furthermore obtained experimentally measured energies of LO phonon modes responsible for Fröhlich interactions in these materials and showed that Fröhlich interactions are higher for bromide perovskites than iodide perovskites, providing a link between composition and electron–phonon scattering that fundamentally limits charge-carrier motion. These results lay the groundwork for more quantitative models of charge-carrier mobility values and cooling dynamics that underpin photovoltaic device operation.

## Methods

### Sample preparation

All materials, unless otherwise stated, were purchased from Sigma-Aldrich and were used as received. Methylammonium iodide, methylammonium bromide, formamidinium iodide and formamidinium bromide were purchased from Dyesol. Thin films were prepared on Z-cut quartz substrates. These were initially cleaned sequentially with acetone followed by propan-2-ol, then treated with oxygen plasma for 10 min.

MA perovskite films were deposited in a nitrogen-filled glovebox using a solvent quenching method wherein an excess of antisolvent is deposited onto the wet substrate while spin-coating^63^. A 1:1 molar ratio solution of MAX and PbX_2_ (X=I, Br) was dissolved in anhydrous *N*,*N*-dimethylformamide at 1 M. This was then spin-coated onto the quartz substrates at 5,000 r.p.m. for 25 s. During spin coating, after 7 s an excess of anhydrous chlorobenzene was rapidly deposited onto the spinning film. After spin-coating, films were annealed at 100 °C for 10 min.

FA perovskite films were deposited using an acid-addition method to produce smooth and uniform pinhole-free films^6^. FAX and PbX_2_ (X=I, Br) were dissolved in anhydrous *N*,*N*-dimethylformamide in a 1:1 molar ratio at 0.55 M. Immediately before film formation, small amounts of acid were added to the precursor solutions, to enhance the solubility of the precursors and allow smooth and uniform film formation. Thirty-eight microlitres of hydroiodic acid (57% mass/mass) was added to 1 ml of the 0.55 M FAPbI_3_ precursor solution and 32 μl of hydrobromic acid (48% mass/mass) was added to 1 ml of the 0.55 M FAPbBr_3_ precursor solution. Films were then spin coated from the precursor plus acid solution on warm (85 °C) oxygen plasma-cleaned substrates at 2,000 r.p.m. in a nitrogen-filled glovebox and subsequently annealed in air at 170 °C for 10 min.

### PL spectroscopy

Each sample was photoexcited by a 398-nm picosecond pulsed diode laser (PicoHarp, LDH-D-C-405M) with a repetition rate of 10 kHz and a fluence of 490 nJ cm^−2^. The resultant PL was collected and focused into a grating spectrometer (Princeton Instruments, SP-2558), which directed the spectrally dispersed PL onto an iCCD (PI-MAX4, Princeton Instruments). The sample was mounted under vacuum (*P*<10^−6^ mbar) in a cold-finger liquid helium cryostat (Oxford Instruments, MicrostatHe). An associated temperature controller (Oxford Instruments, ITC503) monitored the temperature at two sensors mounted on the heat exchanger of the cryostat and the end of the sample holder, respectively; the reading from the latter was taken as the sample temperature. PL measurements were taken as the sample was heated in increments of 5 K between 10 and 370 K.

### Computational methods

We carried out *ab initio* calculations on MAPbI_3_ in the orthorhombic phase using the crystallographic data in ref. 35. The ground-state electronic structure was computed within the local density approximation to DFT including spin–orbit coupling, as implemented in the Quantum ESPRESSO package^64^. We used norm-conserving pseudopotentials to describe the core–valence interaction, with the semicore *d* states taken explicitly into account in the case of Pb and I. Ground-state calculations were converged with a plane-wave cutoff of 100 Ry and a 6 × 6 × 6 unshifted Brillouin-zone grid. The electronic quasiparticle energies were calculated with the SS-*GW* method described in ref. 65 using the Yambo code^66^ and interpolated by means of Wannier functions as in ref. 55, using wannier90 (ref. 67). This yields bandgap and effective masses in good agreement with experiment (see Supplementary Note 1). The lattice dynamical properties were computed within density functional perturbation theory at the Г point, as in ref. 51). The LO–TO splitting was included through the evaluation of the non-analytic contribution to the dynamical matrix. The electron–phonon coupling was calculated using the EPW code^68^,^69^, v.4. The electron–phonon self-energy, *Σ*_*n***k**_, was calculated as:


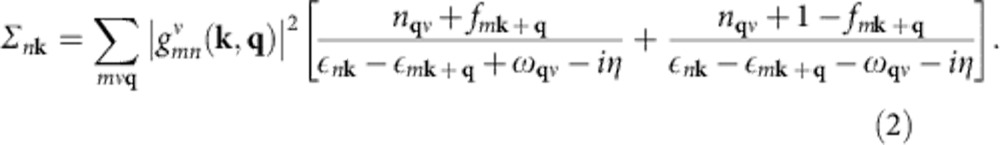


Here, 
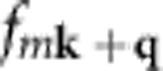
 and *n*_**q***v*_ are the Fermi–Dirac and the Bose–Einstein occupations, respectively, *ɛ*_*n***k**_ and *ɛ*_*m***k+q**_ are electron energies, *ℏω*_**q***v*_ is the energy of a phonon with wavevector **q** and polarization *ν*, and *η* is a small broadening (10 meV in Fig. 3a,b; 1 meV in Fig. 3d). Only the interaction with the LO phonons was taken into account, by calculating the electron–phonon matrix element 
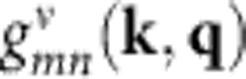
 as in ref. 70 (see Supplementary equation (1)). The self-energy in equation (2) was converged by using up to two million random **q** points in the Brillouin zone. The quasiparticle renormalization factor *Z* is defined as the frequency derivative:


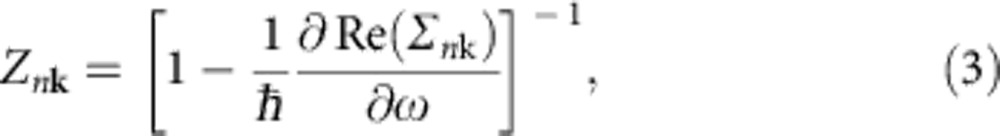


and is evaluated at the band edges. In the case of the conduction band edge, we find *Z*=0.54 at zero temperature.

### Data availability

The data that support the findings of this study are available from the corresponding author on request, as is the custom Matlab code used to analyse the PL data. The Quantum ESPRESSO^64^, Yambo^66^, wannier90 (ref. 67) and EPW v.4 (ref. 69) software used to perform the first-principles calculations are open source and are accessible online.

## Additional information

**How to cite this article:** Wright, A. D. *et al*. Electron–phonon coupling in hybrid lead halide perovskites. *Nat. Commun.* 7:11755 doi: 10.1038/ncomms11755 (2016).

## Supplementary Material

Supplementary Information Supplementary Figures 1-4, Supplementary Tables 1-2, Supplementary Note 1, Supplementary Equation 1 and Supplementary References

## Figures and Tables

**Table 1:** Extracted linewidth parameters.

Sample	*Г*_0_/meV	*γ*_LO_/meV	*E*_LO_/meV
FAPbI_3_	19±1	40±5	11.5±1.2
FAPbBr_3_	20±1	61±7	15.3±1.4
MAPbI_3_	26±2	40±2	*[11.5]*
MAPbBr_3_	32±2	58±2	*[15.3]*

FA, formamidinium; LO, longitudinal optical; MA, methylammonium.

Linewidth broadening parameters extracted from fits of *Г*(*T*)=*Г*_0_+*Г*_LO_ to the PL linewidth data for the four hybrid perovskite films. *Г*_0_ is the inhomogeneous broadening (the linewidth at 0 K), *γ*_LO_ is the strength of the LO phonon–charge-carrier Fröhlich coupling and *E*_LO_ is the relevant LO phonon energy. For fits to the data from MA-containing perovskites, the values of *E*_LO_ extracted previously for the FA-containing perovskites were used (hence, they are italicized and enclosed in square brackets). Because of the additional defect luminescence present in the orthorhombic phase of the MA-containing perovskites, fits were only carried out between 150 and 370 K for MAPbI_3_, and between 100 and 370 K for MAPbBr_3_ (see solid lines in Fig. 2c,d Therefore, the extracted parameters do not reflect the lineshape broadening in the low-temperature phase of the MA-containing perovskites.

## References

[b1] Kojima A., Teshima K., Shirai Y. & Miyasaka T. Organometal halide perovskites as visible-light sensitizers for photovoltaic cells. J. Am. Chem. Soc. 131, 6050–6051 (2009). 19366264 10.1021/ja809598r

[b2] Yang W. S. . High-performance photovoltaic perovskite layers fabricated through intramolecular exchange. Science 348, 1234–1237 (2015). 25999372 10.1126/science.aaa9272

[b3] Bi D. . Efficient luminescent solar cells based on tailored mixed-cation perovskites. Sci. Adv. 2, e1501170 (2016). 26767196 10.1126/sciadv.1501170 PMC4705040

[b4] Herz L. M. Charge carrier dynamics in organic-inorganic metal halide perovskites. Annu. Rev. Phys. Chem. 67, doi:10.1146/annurev-physchem-040215-112222 (2016). 26980309

[b5] McMeekin D. P. . A mixed-cation lead mixed-halide perovskite absorber for tandem solar cells. Science 351, 151–155 (2016). 26744401 10.1126/science.aad5845

[b6] Eperon G. E. . Formamidinium lead trihalide: a broadly tunable perovskite for efficient planar heterojunction solar cells. Energy Environ. Sci. 7, 982–988 (2014).

[b7] Jeon N. J. . Compositional engineering of perovskite materials for high-performance solar cells. Nature 517, 476–480 (2015). 25561177 10.1038/nature14133

[b8] Binek A., Hanusch F. C., Docampo P. & Bein T. Stabilization of the trigonal high temperature phase of formamidinium lead iodide. J. Phys. Chem. Lett. 6, 1249–1253 (2015). 26262982 10.1021/acs.jpclett.5b00380

[b9] Eperon G. E., Beck C. E. & Snaith H. J. Cation exchange for thin film lead iodide perovskite interconversion. Mater. Horiz. 3, 63–71 (2015).

[b10] Rehman W. . Charge-carrier dynamics and mobilities in formamidinium lead mixed-halide perovskites. Adv. Mater. 27, 7938–7944 (2015). 26402226 10.1002/adma.201502969

[b11] Lee M. M., Teuscher J., Miyasaka T., Murakami T. N. & Snaith H. J. Efficient hybrid solar cells based on meso-superstructured organometal halide perovskites. Science 338, 643–647 (2012). 23042296 10.1126/science.1228604

[b12] D'Innocenzo V. . Excitons versus free charges in organo-lead tri-halide perovskites. Nat. Commun. 5, 3586 (2014). 24710005 10.1038/ncomms4586

[b13] Stranks S., Eperon G. & Grancini G. Electron-hole diffusion lengths exceeding 1 micrometer in an organometal trihalide perovskite absorber. Science 342, 341–344 (2013). 24136964 10.1126/science.1243982

[b14] Wehrenfennig C., Eperon G. E., Johnston M. B., Snaith H. J. & Herz L. M. High charge carrier mobilities and lifetimes in organolead trihalide perovskites. Adv. Mater. 26, 1584–1589 (2014). 24757716 10.1002/adma.201305172 PMC4722848

[b15] Johnston M. B. & Herz L. M. Hybrid Perovskites for photovoltaics: charge-carrier recombination, diffusion, and radiative efficiencies. Acc. Chem. Res. 49, 146–154 (2016). 26653572 10.1021/acs.accounts.5b00411

[b16] Zhu X.-Y. & Podzorov V. Charge carriers in hybrid organic-inorganic lead halide perovskites might be protected as large polarons. J. Phys. Chem. Lett. 6, 4758–4761 (2015). 26575427 10.1021/acs.jpclett.5b02462

[b17] Brenner T. M. . Are mobilities in hybrid organic-inorganic halide perovskites actually ‘high'? J. Phys. Chem. Lett. 6, 4754–4757 (2015). 26631359 10.1021/acs.jpclett.5b02390

[b18] Yu P. Y. & Cardona M. Fundamentals of Semiconductors. Graduate Texts in Physics Springer-Verlag (2010).

[b19] Yang Y. . Observation of a hot-phonon bottleneck in lead-iodide perovskites. Nature Photon. 10, 53–59 (2015).

[b20] Wehrenfennig C., Liu M., Snaith H. J., Johnston M. B. & Herz L. M. Homogeneous emission line broadening in the organo lead halide perovskite CH_3_NH_3_PbI_3−*x*_Cl_*x*_. J. Phys. Chem. Lett. 5, 1300–1306 (2014). 26269971 10.1021/jz500434p

[b21] Milot R. L., Eperon G. E., Snaith H. J., Johnston M. B. & Herz L. M. Temperature-dependent charge-carrier dynamics in CH_3_ NH_3_ PbI_3_ perovskite thin films. Adv. Funct. Mater. 25, 6218–6227 (2015).

[b22] Oga H., Saeki A., Ogomi Y., Hayase S. & Seki S. Improved understanding of the electronic and energetic landscapes of perovskite solar cells: high local charge carrier mobility, reduced recombination, and extremely shallow traps. J. Am. Chem. Soc. 136, 13818–13825 (2014). 25188538 10.1021/ja506936f

[b23] Savenije T. . Thermally activated exciton dissociation and recombination control the carrier dynamics in organometal halide perovskite. J. Phys. Chem. Lett. 5, 2189–2194 (2014). 26279532 10.1021/jz500858a

[b24] Karakus M. . Phonon-electron scattering limits free charge mobility in methylammonium lead iodide perovskites. J. Phys. Chem. Lett. 6, 4991–4996 (2015). 26619006 10.1021/acs.jpclett.5b02485

[b25] Seitz F. On the mobility of electrons in pure non-polar insulators. Phys. Rev. 73, 549–564 (1948).

[b26] Bardeen J. & Shockley W. Deformation potentials and mobilities in non-polar crystals. Phys. Rev. 80, 72–80 (1950).

[b27] Benavides-Garcia M. & Balasubramanian K. Bond energies, ionization potentials, and the singlet-triplet energy separations of SnCl_2_, SnBr_2_, SnI_2_, PbCl_2_, PbBr_2_, PbI_2_, and their positive ions. J. Chem. Phys. 100, 2821–2830 (1994).

[b28] Viswanath A., Lee J., Kim D., Lee C. & Leem J. Exciton-phonon interactions, exciton binding energy, and their importance in the realization of room-temperature semiconductor lasers based on GaN. Phys. Rev. B 58, 16333–16339 (1998).

[b29] Stillman G., Wolfe C. & Dimmock J. Hall coefficient factor for polar mode scattering in n-type GaAs. J. Phys. Chem. Solids 31, 1199–1204 (1970).

[b30] Stoumpos C., Malliakas C. & Kanatzidis M. Semiconducting tin and lead iodide perovskites with organic cations: phase transitions, high mobilities, and near-infrared photoluminescent properties. Inorg. Chem. 52, 9019–9038 (2013). 23834108 10.1021/ic401215x

[b31] Fang H.-H. . Photophysics of organic-inorganic hybrid lead iodide perovskite single crystals. Adv. Funct. Mater. 25, 2378–2385 (2015).

[b32] Ha S. T. . Synthesis of organic-inorganic lead halide perovskite nanoplatelets: towards high-performance perovskite solar cells and optoelectronic devices. Adv. Opt. Mater. 2, 838–844 (2014).

[b33] Fang H. H. . Photoexcitation dynamics in solution-processed formamidinium lead iodide perovskite thin films for solar cell applications. Light Sci. Appl. 5, e16056 (2016). 10.1038/lsa.2016.56 PMC6059954 30167155

[b34] Onoda-Yamamuro N., Matsuo T. & Suga H. Calorimetric and IR spectroscopic studies of phase transitions in methylammonium trihalogenoplumbates (II). J. Phys. Chem. Solids 51, 1383–1395 (1990).

[b35] Baikie T. . Synthesis and crystal chemistry of the hybrid perovskite (CH_3_NH_3_)PbI_3_ for solid-state sensitised solar cell applications. J. Mater. Chem. A 1, 5628–5641 (2013).

[b36] Wasylishen R., Knop O. & Macdonald J. Cation rotation in methylammonium lead halides. Solid State Commun. 56, 581–582 (1985).

[b37] Varshni Y. Temperature dependence of the energy gap in semiconductors. Physica 34, 149–154 (1967).

[b38] Frost J. M. J. . Atomistic origins of high-performance in hybrid halide perovskite solar cells. Nano Lett. 14, 2584–2590 (2014). 24684284 10.1021/nl500390f PMC4022647

[b39] Wehrenfennig C., Liu M., Snaith H. J., Johnston M. B. & Herz L. M. Charge carrier recombination channels in the low-temperature phase of organic-inorganic lead halide perovskite thin films. APL Mater 2, 081513 (2014).

[b40] Wu X. . Trap states in lead iodide perovskites. J. Am. Chem. Soc. 137, 2089–2096 (2015). 25602495 10.1021/ja512833n

[b41] Priante D. . The recombination mechanisms leading to amplified spontaneous emission at the true-green wavelength in CH_3_NH_3_PbBr_3_ perovskites. Appl. Phys. Lett. 106, 081902 (2015).

[b42] Rudin S., Reinecke T. L. & Segall B. Temperature-dependent exciton linewidths in semiconductors. Phys. Rev. B 42, 11218–11231 (1990). 10.1103/physrevb.42.11218 9995407

[b43] Lee J., Koteles E. S. & Vassell M. O. Luminescence linewidths of excitons in GaAs quantum wells below 150 K. Phys. Rev. B 33, 5512–5516 (1986). 10.1103/physrevb.33.5512 9939057

[b44] Malikova L. . Temperature dependence of the direct gaps of ZnSe and Zn_0.56_Cd_0.44_Se. Phys. Rev. B 54, 1819–1824 (1996). 10.1103/physrevb.54.1819 9986029

[b45] Chen Y., Kothiyal G., Singh J. & Bhattacharya P. Absorption and photoluminescence studies of the temperature dependence of exciton life time in lattice-matched and strained quantum well systems. Superlattice Microst. 3, 657–664 (1987).

[b46] Bartolo B. D. & Chen X. Advances in Energy Transfer Processes World Scientific (2001).

[b47] Selci S. . Evaluation of electron-phonon coupling of Al_0.27_Ga_0.73_As/GaAs quantum wells by normal incidence reflectance. Solid State Commun. 79, 561–565 (1991).

[b48] Masumoto Y. & Takagahara T. Semiconductor Quantum Dots Springer-Verlag (2002).

[b49] Zhang X. B., Taliercio T., Kolliakos S. & Lefebvre P. Influence of electron-phonon interaction on the optical properties of III nitride semiconductors. J. Phys.: Condens. Matter 13, 7053–7074 (2001).

[b50] Gammon D., Rudin S., Reinecke T. L., Katzer D. S. & Kyono C. S. Phonon broadening of excitons in GaAs/AlGaAs quantum wells. Phys. Rev. B 51, 16785–16789 (1995). 10.1103/physrevb.51.16785 9978686

[b51] Pérez-Osorio M. A. . Vibrational properties of the organic-inorganic halide perovskite CH_3_NH_3_PbI_3_ from theory and experiment: factor group analysis, first-principles calculations, and low-temperature infrared spectra. J. Phys. Chem. C 119, 25703–25718 (2015).

[b52] Yamada Y. . Photocarrier recombination dynamics in perovskite CH_3_NH_3_PbI_3_ for solar cell applications. J. Am. Chem. Soc. 136, 11610–11613 (2014). 25075458 10.1021/ja506624n

[b53] Gopalan S., Lautenschlager P. & Cardona M. Temperature dependence of the shifts and broadenings of the critical points in GaAs. Phys. Rev. B 35, 5577–5584 (1987). 10.1103/physrevb.35.5577 9940769

[b54] Grimvall G. The Electron-Phonon Interaction in Metals North-Holland (1981).

[b55] Filip M. R., Verdi C. & Giustino F. *GW* band structures and carrier effective masses of CH_3_NH_3_PbI_3_ and hypothetical perovskites of the type APbI_3_: A=NH_4_, PH_4_, AsH_4_, and SbH_4_. J. Phys. Chem. C 119, 25209–25219 (2015).

[b56] Wehrenfennig C., Liu M., Snaith H. J., Johnston M. B. & Herz L. M. Charge-carrier dynamics in vapour-deposited films of the organolead halide perovskite CH_3_NH_3_PbI_3*−x*_Cl_*x*_. Energy Environ. Sci. 7, 2269–2275 (2014).

[b57] Petritz R. & Scanlon W. Mobility of electrons and holes in the polar crystal, PbS. Phys. Rev. 97, 1620–1626 (1955).

[b58] Ferry D. First-order optical and intervalley scattering in semiconductors. Phys. Rev. B 14, 1605–1609 (1976).

[b59] Price M. . Hot carrier cooling and photo-induced refractive index changes in organic-inorganic lead halide perovskites. Nat. Commun. 6, 8420 (2015). 26404048 10.1038/ncomms9420 PMC4598728

[b60] Kawai H., Giorgi G., Marini A. & Yamashita K. The mechanism of slow hot-hole cooling in lead-iodide perovskite: first-principle calculation on carrier lifetime from electron-phonon interaction. Nano Lett. 15, 3103–3108 (2015). 25807270 10.1021/acs.nanolett.5b00109

[b61] von der Linde D. & Lambrich R. Direct measurement of hot-electron relaxation by picosecond spectroscopy. Phys. Rev. Lett. 42, 1090–1093 (1979).

[b62] Sadhanala A. . Preparation of single phase films of CH_3_NH_3_Pb(I_1−*x*_Br_*x*_)_3_ with sharp optical band edges. J. Phys. Chem. Lett. 5, 2501–2505 (2014). 26277936 10.1021/jz501332v

[b63] Xiao M. . A fast deposition-crystallization procedure for highly efficient lead iodide perovskite thin-film solar cells. Angew. Chem. Int. Ed. 126, 10056–10061 (2014). 10.1002/anie.201405334 25047967

[b64] Giannozzi P. . QUANTUM ESPRESSO: a modular and open-source software project for quantum simulations of materials. J. Phys.: Condens. Matter 21, 395502 (2009). 21832390 10.1088/0953-8984/21/39/395502

[b65] Filip M. R. & Giustino F. GW quasiparticle band gap of the hybrid organic-inorganic perovskite CH_3_NH_3_PbI_3_: Effect of spin-orbit interaction, semicore electrons, and self-consistency. Phys. Rev. B 90, 245145 (2014).

[b66] Marini A., Hogan C., Grüning M. & Varsano D. yambo: An *ab initio* tool for excited state calculations. Comp. Phys. Commun. 180, 1392–1403 (2009).

[b67] Mostofi A. A. . wannier90: A tool for obtaining maximally-localised Wannier functions. Comp. Phys. Commun. 178, 685–699 (2008).

[b68] Giustino F., Cohen M. L. & Louie S. G. Electron-phonon interaction using Wannier functions. Phys. Rev. B 76, 165108 (2007). 10.1103/PhysRevLett.98.047005 17358802

[b69] Poncé S., Margine E. R., Verdi C. & Giustino F. EPW: electron-phonon coupling, transport and superconducting properties using maximally localized Wannier functions. Preprint at http://arxiv.org/abs/1604.03525 (2016).

[b70] Verdi C. & Giustino F. Fröhlich electron-phonon vertex from first principles. Phys. Rev. Lett. 115, 176401 (2015). 26551127 10.1103/PhysRevLett.115.176401

