# Supplementary material for: Electron–phonon coupling in hybrid lead halide perovskites
Source: Nat Commun. 2016 May 26;7:0. doi: 10.1038/ncomms11755 (PMC4894981; doi:10.1038/ncomms11755)
Supplement: Supplementary Information — Supplementary Figures 1-4, Supplementary Tables 1-2, Supplementary Note 1, Supplementary Equation 1 and Supplementary References [file ncomms11755-s1.pdf]

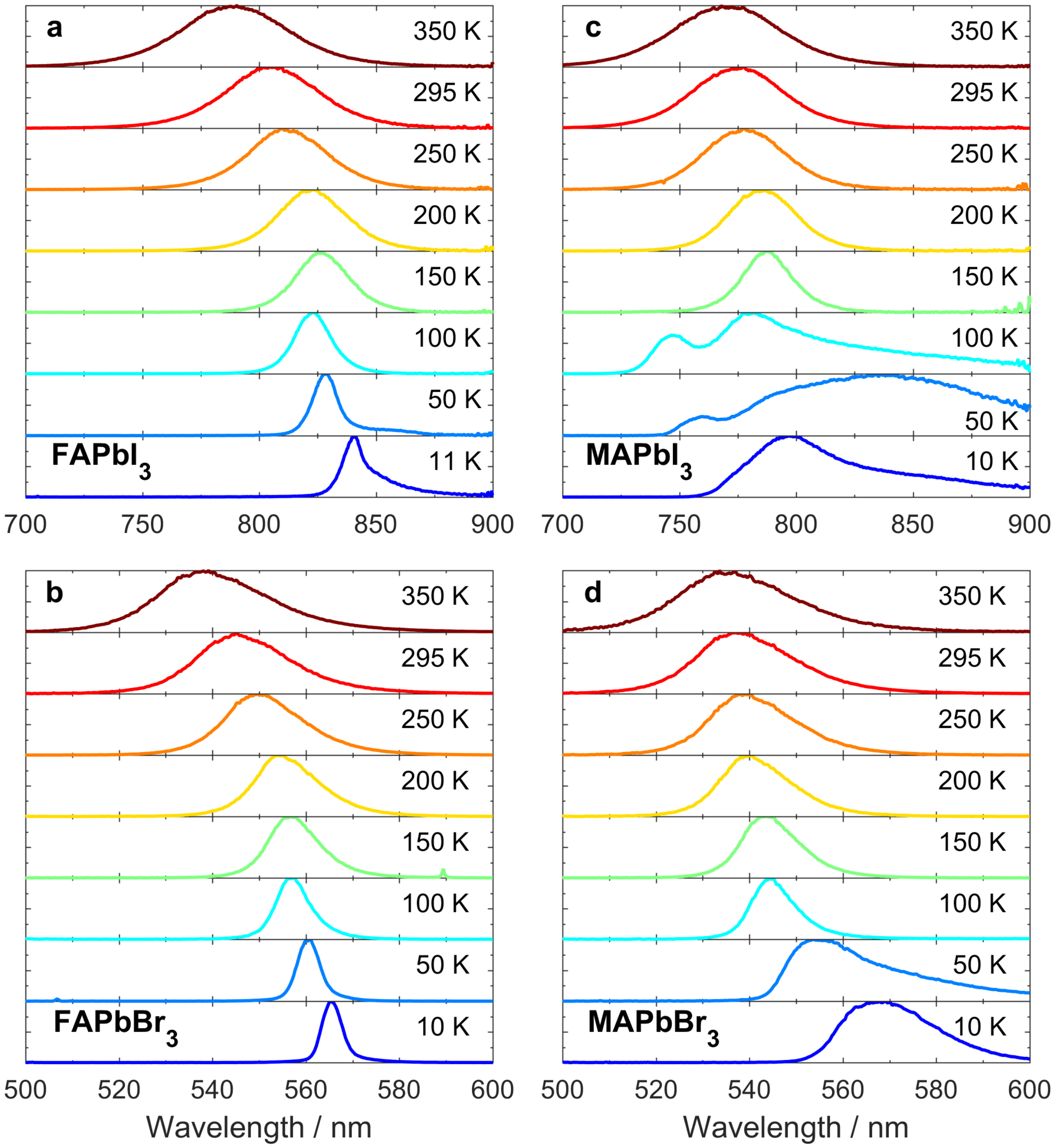

Supplementary Figure 1 | PL spectra at selected temperatures. Steady-state PL spectra at selected temperatures for **(a)** $FAPbI_3$, **(b)** $FAPbBr_3$, **(c)** $MAPbI_3$ and **(d)** $MAPbBr_3$. The y-direction represents the normalised PL intensity.

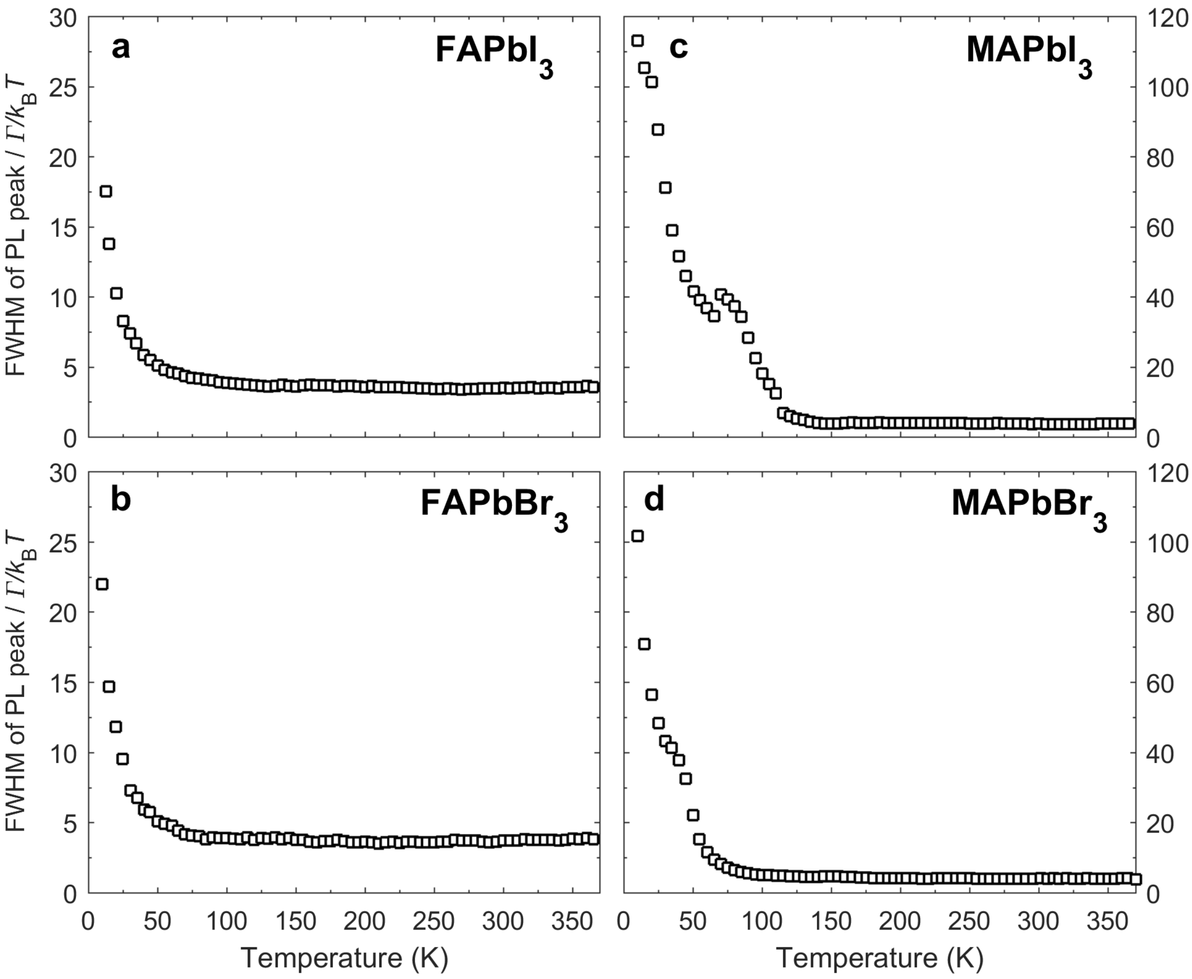

Supplementary Figure 2 | Temperature variation of PL linewidth as a multiple of thermal energy. Temperature dependence of the full width at half maximum (FWHM, plotted as black squares) of the steady-state PL spectra expressed as a multiple of the thermal energy k_BT, for **(a)** $FAPbI_3$, **(b)** $FAPbBr_3$, **(c)** $MAPbI_3$ and **(d)** $MAPbBr_3$.

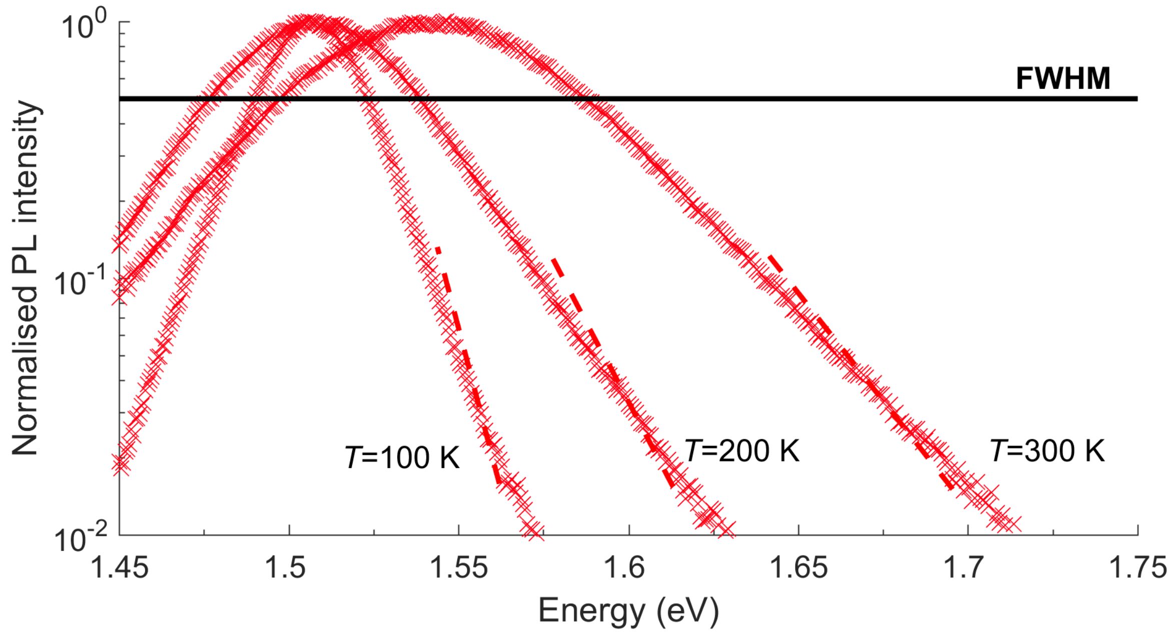

Supplementary Figure 3 | Carrier energy distribution from PL tails. Steady-state PL spectra at temperatures (T) for an $FAPbI_3$ thin film, plotted as red crosses. The high-energy tails of the band edge PL can be approximated by the dashed red lines representing the functional form $I(E) \propto exp\left[-(E - E_{\mathrm{g}})/k_{\mathrm{B}}T_{\mathrm{c}}\right]$, which describes the PL lineshape expected from thermalized charge carriers of temperature T_{c} following a Boltzmann energy distribution[1]. I is the PL intensity, E is the PL energy and E_{g} is the bandgap energy. The solid black line indicates the position on the curves at which the FWHM is measured, which lies above the high-energy tail of the band edge PL. For the shown dashed red lines, the carrier temperature T_{c} was set as the sample temperature T. However, slightly shallower tails (indicating T_{c} somewhat higher than T) are actually observed. One reason for this discrepancy may lie in the strongly non-resonant excitation at 3.1 eV which leads to an initial thermalized "hot" charge-carrier density that subsequently cools to the lattice temperature[2,3]. Since the PL curves shown represent an average over the charge-carrier temperature during the lifetime of charge-carriers, a temperature slightly elevated above the lattice temperature may be found. However, the overall line shape is also strongly governed by electron-phonon coupling, as described in the main text, whose effects will influence the spectral shape also in the high-energy tail.

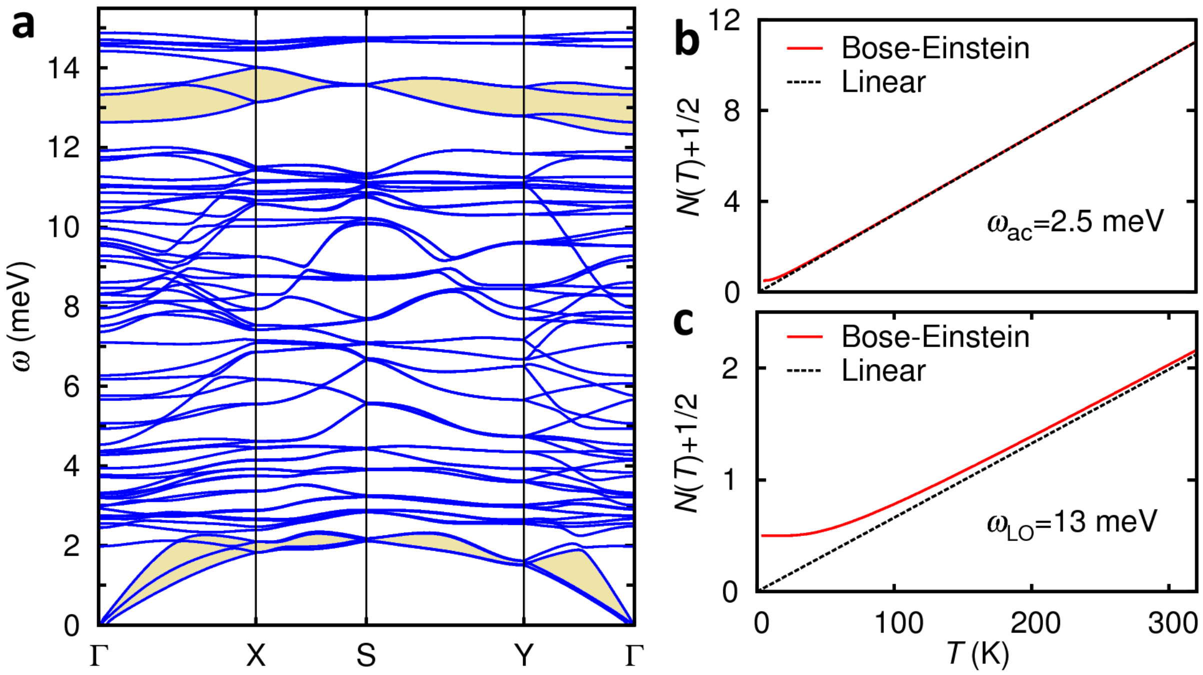

Supplementary Figure 4 | Phonon dispersion and population relations from *ab initio* calculations. The calculated phonon dispersion relation for $MAPbI_3$ is shown in **(a)**, with the acoustic modes (bottom) and the polar optical modes (top) highlighted in orange. Here we see that the frequency of the acoustic modes is always smaller than 2.5 meV, while the polar optical modes are centred on 13 meV. On the right, the phonon population according to the Bode-Einstein function, $N(T)$, is plotted in red as a function of temperature for **(b)** the acoustic modes and **(c)** the polar optical modes. The Bose-Einstein phonon population is offset vertically by +½ to allow direct comparison with linear approximations to the phonon population, which are plotted as dotted black lines. The linear temperature dependence used in Eqn. 1 in the main text for the linewidth broadening due to acoustic phonons is therefore very accurate in the case of $MAPbI_3$ for all temperatures above 10 K, while the Bose-Einstein function is necessary to model the broadening due to optical phonons.

Supplementary Table 1 | Linewidth parameters for perovskites and inorganic semiconductors. Collated values of linewidth parameters for inorganic semiconductors reported by other experimental studies, along with those obtained from our data. We use the FWHM definition of linewidth, but some papers, indicated by an asterisk (*) in the materials column, define it as the HWHM (Half Width at Half Maximum); we multiply by two the Γ_0, γ_{ac}, γ_{LO} and γ_{imp} values from these papers. Blank cells indicate parameters which were not considered in a paper. When a parameter used in a fit was fixed, that value is underlined in the table. Materials were studied in the bulk, except for those at the bottom of the table, which were studied as quantum wells (indicated by §), or epilayers (‡).

Material	Technique	Source	Γ_0 (meV)	γ_{ac} (μeV K^{-1})	γ_{LO} (meV)	E_{LO} (meV)	γ_{imp} (meV)	E_b (meV)
**$FAPbI_3$**	PL		19±1		40±5	11.5±1.2		
**$FAPbBr_3$**	PL		20±1		61±7	15.3±1.4		
**$MAPbI_3$**	PL		26±2		40±2	11.5		
**$MAPbBr_3$**	PL		32±2		58±2	15.3		
**CdS**	A	[4]*	0.32	29.0	212	38		
**CdSe**	E	[5]†*	4.6±0.6		46±2	25.9		
**CdTe**	R	[6]*			34±14			
**GaAs**	PL	[7]	0.32	13±3	30.4±4			
	A	[8]*		14	17.4	36.6		
	R	[9]*			40±2	36		
**ZnO**	PL	[10]	10.3		962.7	71	13.8	9.2
**ZnSe**	R	[6]*			60±14			
	FWM	[11]	1.3	11	81			
**$CdTe/Cd_{1-x}Mn_xTe$**	FWM	[12]§	0.89±0.05	13.7±1.8				
**$CdTe/Cd_{1-x}Zn_xTe$**	PL	[13]§	75		400	22	500	92
**$GaAs/Al_xGa_{1-x}As$**	PL	[14]§ HH *	0.9	2.94	8	36	1.5	10
	PL	[14]§ LH *	0.6	2.38	4.9	36	0.4	10
**GaN**	PL	[15]‡†*	5.6	42	1050	91.5		
**$Zn_{0.56}Cd_{0.44}Se$**	R	[16]‡*	12±4	2.2	34±12	28.8		
**ZnSe**	R	[16]‡*	13±5	4.0	48±16	31		

Key to superscripts:

A	Absorption
E	Ellipsometry
FWM	Four-wave mixing
PL	Photoluminescence
R	Reflectance
*	Original linewidth stated in terms of HWHM
†	Linewidth of exciton A
HH	Heavy hole exciton
LH	Light hole exciton
§	Quantum well
‡	Epilayer

Supplementary Table 2 | Calculated electrical parameters. Calculated Born effective charges (Z^*), high-frequency and static dielectric constants (ϵ^{∞} and ϵ^0 respectively) of $MAPbI_3$ and $MAPbBr_3$ in the orthorhombic phase. We report the isotropic average of the tensors, and we compare the dielectric constants to experiment.

	Z^*				ϵ			
	Pb	I/Br	C	N	ϵ^{∞}	$\epsilon^{\infty}_{\text{exp}}$	ϵ^0	ϵ^0_{exp}
$MAPbI_3$	+4.42	-1.88	+0.03	-0.95	5.86	6.5[17]	25.3	30.5[18]
$MAPbBr_3$	+4.11	-1.78	+0.09	-0.80	4.71	4.8[19]	23.9	26.2[18]

Supplementary Note 1 | Details of first-principles calculations. The GW calculations for the quasiparticle energies were performed as described previously[20]. The SS-GW eigenvalues were interpolated using Wannier functions starting from a $4\times4\times4$ grid. This gives effective masses in very good agreement with the experiment, which is crucial for electron-phonon calculations. In fact, the electron-phonon self-energy at the band edges is very sensitive to the curvature of the bands.

The lattice dynamical properties of $MAPbI_3$ were calculated according to a previously published procedure[21]. All calculations were performed for the low-temperature orthorhombic structure of $MAPbI_3$, since the vibrational frequencies are relatively insensitive to the structure[21], and the measured PL broadening does not change across the orthorhombic/tetragonal phase transition. In the case of $MAPbBr_3$ the vibrational properties were calculated for the orthorhombic structure, starting from the lattice parameters and atomic coordinates given by Swainson *et al.*[22].

In order to evaluate the electron-phonon self-energy we calculated the electron-phonon matrix elements using the *ab initio* Fröhlich vertex[23] in its simplified form, as given in Supplementary Equation 1. The calculated Born effective charges and dielectric constants are reported in Supplementary Table 2. Additionally, we accounted for the quasiparticle renormalization of the electron lifetime by calculating the factor Z, which corresponds to an improved solution of the Dyson equation[24,25].

Supplementary Equation 1 | *Ab initio* Fröhlich vertex. Here $\mathbf{Z}^* = Z^*_{\alpha\beta}$ is the Born effective charge tensor, $\boldsymbol{\epsilon}_\infty = \epsilon_{\infty,\alpha\beta}$ is the high-frequency dielectric tensor, $\mathbf{e}_{\kappa\nu}(\mathbf{q})$ is a vibrational eigenmode, $\mathbf{G}$ represents a reciprocal lattice vector, and M_κ is the mass of atom κ.

$$g^{\nu}_{mn}(\mathbf{q}) = i\,\frac{4\pi e^2}{\Omega}\sum_{\kappa}\left(\frac{\hbar}{2M_\kappa\omega_{\mathrm{q}\nu}}\right)^{\frac{1}{2}}\sum_{\mathbf{G}\neq-\mathbf{q}}\frac{(\mathbf{q}+\mathbf{G})\cdot\mathbf{Z}^*_\kappa\cdot\mathbf{e}_{\kappa\nu}(\mathbf{q})}{(\mathbf{q}+\mathbf{G})\cdot\boldsymbol{\epsilon}^{\infty}\cdot(\mathbf{q}+\mathbf{G})}\,\delta_{mn} \qquad (1)$$

Supplementary References

1. Zhang, X. B., Taliercio, T., Kolliakos, S. & Lefebvre, P. Influence of electron-phonon interaction on the optical properties of III nitride semiconductors. *J. Phys. Condens. Matter* **13,** 7053–7074 (2001).
2. Price, M. *et al.* Hot carrier cooling and photo-induced refractive index changes in organic-inorganic lead halide perovskites. *Nat. Commun.* **6,** 8420 (2015).
3. Yang, Y. *et al.* Observation of a hot-phonon bottleneck in lead-iodide perovskites. *Nat. Photon.* **10,** 53–59 (2015).
4. Spiegelberg, F., Gutsche, E. & Voigt, J. Exciton-phonon interaction in CdS. *Phys. Status Solidi B* **77,** 233–242 (1976).
5. Logothetidis, S., Cardona, M., Lautenschlager, P. & Garriga, M. Temperature dependence of the dielectric function and the interband critical points of CdSe. *Phys. Rev. B* **34,** 2458–2469 (1986).
6. Rudin, S., Reinecke, T. L. & Segall, B. Temperature-dependent exciton linewidths in semiconductors. *Phys. Rev. B* **42,** 11218–11231 (1990).
7. Gopal, A. V. *et al.* Photoluminescence study of exciton–optical phonon scattering in bulk GaAs and GaAs quantum wells. *J. Appl. Phys.* **87,** 1858–1862 (2000).
8. Gammon, D., Rudin, S., Reinecke, T. L., Katzer, D. S. & Kyono, C. S. Phonon broadening of excitons in GaAs/AlGaAs quantum wells. *Phys. Rev. B* **51,** 16785–16789 (1995).
9. Qiang, H. *et al.* Size dependence of the thermal broadening of the exciton linewidth in GaAs/$Ga_{0.7}Al_{0.3}As$ single quantum wells. *Appl. Phys. Lett.* **61,** 1411 (1992).
10. Fonoberov, V. A., Alim, K. A., Balandin, A. A., Xiu, F. & Liu, J. Photoluminescence investigation of the carrier recombination processes in ZnO quantum dots and nanocrystals. *Phys. Rev. B* **73,** 165317 (2006).
11. Fischer, A. *et al.* Femtosecond coherent spectroscopy of bulk ZnSe and ZnCdSe/ZnSe quantum wells. *Phys. Rev. Lett.* **73,** 2368–2371 (1994).
12. Hellmann, R. *et al.* Homogeneous linewidth of excitons in semimagnetic CdTe/$Cd_{1-x}Mn_xTe$ multiple quantum wells. *Physical Review B* **48,** 2847–2850 (1993).
13. Li, T., Lozykowski, H. & Reno, J. Optical properties of CdTe/$Cd_{1-x}Zn_xTe$ strained-layer single quantum wells. *Phys. Rev. B* **46,** 6961–6968 (1992).
14. Lee, J., Koteles, E. S. & Vassell, M. O. Luminescence linewidths of excitons in GaAs quantum wells below 150 K. *Phys. Rev. B* **33,** 5512–5516 (1986).
15. Viswanath, A., Lee, J., Kim, D., Lee, C. & Leem, J. Exciton-phonon interactions, exciton binding energy, and their importance in the realization of room-temperature semiconductor lasers based on GaN. *Phys. Rev. B* **58,** 16333–16339 (1998).
16. Malikova, L. *et al.* Temperature dependence of the direct gaps of ZnSe and $Zn_{0.56}Cd_{0.44}Se$. *Phys. Rev. B* **54,** 1819–1824 (1996).
17. Hirasawa, M., Ishihara, T., Goto, T., Uchida, K. & Miura, N. Magnetoabsorption of the lowest exciton in perovskite-type compound $(CH_3NH_3)PbI_3$. *Physica B* **201,** 427–430 (1994).
18. Poglitsch, A. & Weber, D. Dynamic disorder in methylammoniumtrihalogenoplumbates (II) observed by millimeter-wave spectroscopy. *J. Chem. Phys.* **87,** 6373–6378 (1987).
19. Tanaka, K. *et al.* Comparative study on the excitons in lead-halide-based perovskite-type crystals $CH_3NH_3PbBr_3$ CH3NH3PbI_3. *Solid State Commun.* **127,** 619–623 (2003).
20. Filip, M. R., Verdi, C. & Giustino, F. *GW* band structures and carrier effective masses of $CH_3NH_3PbI_3$ and hypothetical perovskites of the type $APbI_3$: A = NH_4, PH_4, AsH_4, and SbH_4. *J. Phys. Chem. C* **119,** 25209–25219 (2015).
21. Perez Osorio, M. A. *et al.* Vibrational properties of the organic-inorganic halide perovskite $CH_3NH_3PbI_3$ from theory and experiment: factor group analysis, first-principles calculations, and low-temperature infrared spectra. *J. Phys. Chem. C* **119,** 25703–25718 (2015).
22. Swainson, I. P., Hammond, R. P., Soullière, C., Knop, O. & Massa, W. Phase transitions in the

perovskite methylammonium lead bromide, $CH_3ND_3PbBr_3$. *J. Solid State Chem.* **176,** 97–104 (2003).

23. Verdi, C. & Giustino, F. Fröhlich electron-phonon vertex from first principles. *Phys. Rev. Lett.* **115,** 176401 (2015).
24. Grimvall, G. *The Electron-Phonon Interaction in Metals*. (North-Holland, 1981).
25. Eiguren, A., Ambrosch-Draxl, C. & Echenique, P. M. Self-consistently renormalized quasiparticles under the electron-phonon interaction. *Phys. Rev. B* **79,** 245103 (2009).
